# Enhancing and controlling valley magnetic response in MoS_2_/WS_2_ heterostructures by all-optical route

**DOI:** 10.1038/s41467-019-12128-2

**Published:** 2019-09-17

**Authors:** Jing Zhang, Luojun Du, Shun Feng, Run-Wu Zhang, Bingchen Cao, Chenji Zou, Yu Chen, Mengzhou Liao, Baile Zhang, Shengyuan A. Yang, Guangyu Zhang, Ting Yu

**Affiliations:** 1 Division of Physics and Applied Physics, School of Physical and Mathematical Sciences, Nanyang Technological University, Singapore, 63737 Singapore; 2 CAS Key Laboratory of Nanoscale Physics and Devices, Institute of Physics, Chinese Academy of Sciences, 100190 Beijing, China; 3 Department of Electronics and Nanoengineering, Aalto University, FI-02150 Tietotie 3, Finland; 4 Research Laboratory for Quantum Materials, Singapore University of Technology and Design, Singapore, 487372 Singapore; 5 Key Lab of advanced optoelectronic quantum architecture and measurement (MOE), Beijing Key Lab of Nanophotonics & ultrafine Optoelectronic Systems, and School of Physics, Beijing Institute of Technology, 100081 Beijing, China; 6 Songshan Lake Materials Laboratory, Dongguan, 523808 Guangdong, China

**Keywords:** Two-dimensional materials, Magneto-optics

## Abstract

Van der Waals heterostructures of transition metal dichalcogenides with interlayer coupling offer an exotic platform to realize fascinating phenomena. Due to the type II band alignment of these heterostructures, electrons and holes are separated into different layers. The localized electrons induced doping in one layer, in principle, would lift the Fermi level to cross the spin-polarized upper conduction band and lead to strong manipulation of valley magnetic response. Here, we report the significantly enhanced valley Zeeman splitting and magnetic tuning of polarization for the direct optical transition of MoS_2_ in MoS_2_/WS_2_ heterostructures. Such strong enhancement of valley magnetic response in MoS_2_ stems from the change of the spin-valley degeneracy from 2 to 4 and strong many-body Coulomb interactions induced by ultrafast charge transfer. Moreover, the magnetic splitting can be tuned monotonically by laser power, providing an effective all-optical route towards engineering and manipulating of valleytronic devices and quantum-computation.

## Abstract (web summary)

Van der Waals heterostructures may offer a suitable platform for all-optical manipulation of valleytronic devices. Here, the authors observe a strong enhancement of the valley magnetic response in MoS_2_, and magnetic tuning of the polarization of MoS_2_ direct optical transition

## Introduction

The investigation and control of the valley degree of freedom (DoF) are important topics in condensed matter physics and would give rise to new paradigms to encode and process information for future valleytronic and optoelectronic applications^1–4^. Two-dimensional (2D) transition metal dichalcogenides (TMDCs) with unique electronic band structure, such as direct band gap^5,6^, giant spin-orbit coupling (SOC), and entangled valley and spin DoF^7,8^, provide an unprecedented platform to manipulate the valley pseudospin through circularly polarized light excitation^9–12^ and electric field as well^13–17^. Moreover, due to valley-contrasting Berry curvature and magnetic moment^8^, magnetic field offers an effective opportunity to engineer the valley DoF. Recently, the valley degeneracy in TMDCs has been lifted via applying an external magnetic field, which is known as the valley Zeeman effect and plays a prominent role to manipulate valley polarization and valley coherence for logic valleytronic devices^18–26^. However, the valley splitting reported so far is relatively small, e.g. ~0.23 meV T^–1^, corresponding to a Lande *g* factor around 4^18,24,26–28^. Such small valley splitting values strongly impede the practical application and development of valleytronics since magnetic control is almost impossible for a small external field.

In order to promote the information encoding and quantum-computing applications with valley DoF, great experimental efforts have been invested to enhance the valley splitting. An effective approach is to take advantage of an interfacial magnetic exchange field (MEF) from a ferromagnetic substrate^29,30^. Valley splitting with more than one order of magnitude enhancement is demonstrated in monolayer WSe_2_ by utilizing the proximity interfacial MEF from the ferromagnetic EuS or CrI_3_ substrate^31–34^. Another exotic way is doping which would induce the paramagnetic response of 2D Dirac fermions in TMDCs^35^ as proposed theoretically by T. Cai et al. in 2013. Recently, a giant and tunable valley exciton splitting induced by the strong electron-electron exchange interaction has also been uncovered in a wealth of atomically thin TMDCs through electrostatic gating^21,25,36–40^. In close analogy to electrostatic doping, ultrafast charge transfer in hetero-bilayers of TMDCs with type II band alignments, e.g., MoS_2_/WS_2_ heterostructures, generates electrons and holes separated in different layers and gives rise to the *n*-doping (*p*-doping) for MoS_2_ (WS_2_)^41,42^. Such ultrafast charge transfer induced doping would strengthen the Coulomb exchange interactions, leading to the strongly enhanced valley splitting and susceptibility. However, the greatly improved valley magnetic response has not yet been demystified in any heterostructures of TMDCs. Moreover, the efficient separation of holes and electrons guarantees the possibility to tune the doping concentration by the power of laser, providing an all-optical strategy to control magnetic related spin-valley phenomena^43^. In addition, monolayer TMDCs have short valley lifetime, which severely constrains their practical application^44,45^. In stark contrast, due to ultrafast charge separation in both real space and momentum space, the electron-hole exchange interaction is strongly suppressed in hetero-bilayers. Thus, heterostructures of TMDCs harbor ultra-long valley lifetime and are more promising for valleytronic applications^46–48^. Measurements of the valley magnetic response in heterostructures of TMDCs, brook no delay, which would provide a firm basis for the development of magnetic manipulation of valley DoF.

Here we demonstrate the robust giant magnetic valley splitting of MoS_2_ in as-grown MoS_2_/WS_2_ heterostructures via circular polarization-resolved magneto-photoluminescence measurements. The large enhancement of magnetic response in MoS_2_ is attributed to the significantly enhanced strength of exchange interaction induced by ultrafast charge transfer, which leads to the Fermi level cross the spin-split upper conduction band. Furthermore, the enhanced Zeeman splitting of MoS_2_ exciton emission can be photo-controlled by varying the excitation power from 6.6 to 3.8 meV at 7 T, corresponding to *g* factor from 15.4 to 9.5. The large *g* factor reveals the strong interaction effects in the conduction band of MoS_2_ in MoS_2_/WS_2_ heterostructures. Meanwhile, we further demonstrate that the strongly enhanced valley splitting in MoS_2_/WS_2_ heterostructures make it possible to manipulate the valley polarization effectively through magnetic fields. Our results will stimulate the widespread development in general doping enhanced magnetic susceptibility of 2D electron gas system in van der Waals (vdW) heterostructures.

## Results

### Growth and characterization of MoS_2_/WS_2_ heterostructures

**Fig. 1:**
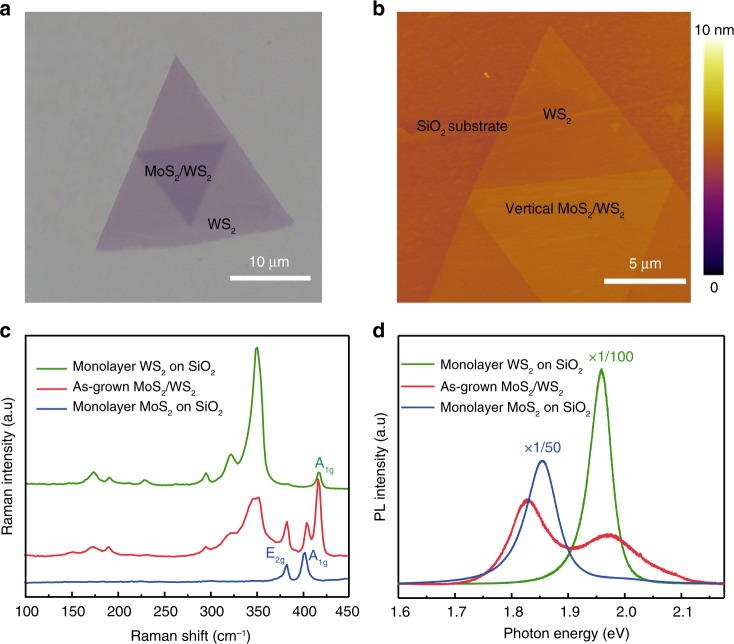
Characterization for vertically stacked MoS_2_/WS_2_ heterostructures. **a** Optical image of hetero-bilayer MoS_2_/WS_2_. **b** Surface morphology illustrated by AFM image. **c**, **d** Room temperature Raman and PL spectra for monolayer WS_2_, MoS_2_/WS_2_, and MoS_2_ respectively

Our high-quality vertical heterostructure samples were synthesized directly on SiO_2_ substrates following two-step process of chemical vapor deposition (CVD)^49^. Initially, well-defined monolayer triangular WS_2_ were grown on 300-nm-thick SiO_2_/Si substrates. Then, using WS_2_ as the growth template, the top layer MoS_2_ with typical lateral size up to 10 μm were synthesized either with parallel or antiparallel stacking, as illustrated in Fig. 1a (See Method for more growth details). The atomically smooth surface of vertically stacked MoS_2_/WS_2_ heterostructures is shown by atomic force microscopy (AFM) image in Fig. 1b. Furthermore, Raman characterization (Fig. 1c) confirms that the top layer is monolayer MoS_2_ with typical in-plane and out-of-plane phonon vibration modes located at 385 cm^−1^ and 404.4 cm^−1^, while the center triangular region contains the Raman modes of both MoS_2_ and WS_2_. Figure 1d shows the room temperature PL for monolayer MoS_2_, WS_2_, and hetero-bilayers. The prominent peak at 1.85 eV (1.96 eV) corresponds to excitonic emission of monolayer MoS_2_ (WS_2_). For epitaxial heterostructures, the profile is dominated by the intralayer exciton of both monolayers with PL intensity quench by a factor of 50 (100) compared with MoS_2_ (WS_2_) which is attributed to efficient interlayer charge transfer between layers^41,42^. Note that interlayer exciton stemmed from the recombination of separated electrons and holes can also be observed at low energy regime from room temperature PL spectra (please refer to Supplementary Fig. 5 for more information). Up to now, interlayer excitons have sparked significant attention due to the fascinating physical properties^50–52^. However, only a few papers focused on the intralayer excitons in the heterostructures which would also possess excellent exotic phenomena and play a key role for valleytronics. In this work, we study the intralayer excitons of MoS_2_/WS_2_ heterostructures to demonstrate the interesting spin-valley related phenomena and their potential applications instead of interlayer excitons.

### First-principles calculations of MoS_2_/WS_2_ heterostructures

**Fig. 2:**
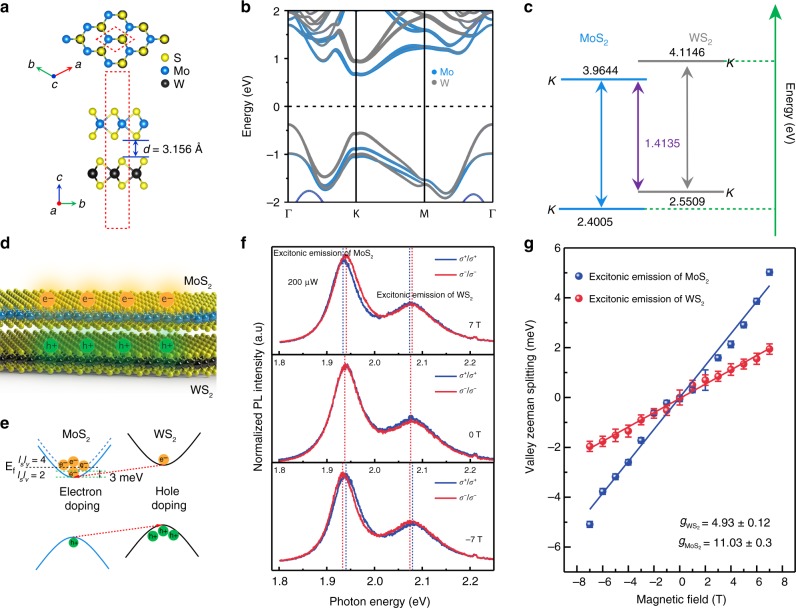
Valley Zeeman splitting for MoS_2_/WS_2_ heterostructures. **a** Top and side view of the atomic structure of MoS_2_/WS_2_ heterostructures. **b** Calculated band structure of MoS_2_/WS_2_ heterostructures with orbital analysis in presence of SOC. The size of the blue (gray) dots denotes the weight of projection onto the Mo-4*d* (W-5*d*) orbitals. **c** Band alignment diagram is schematically depicted with theoretical band gap values for the MoS_2_ and WS_2_ monolayers. **d** Electrons and holes separated in different layers under optical excitation. **e** Schematic image for electronic band structures of electron doping induced fermi level lifting from spin-valley degeneracy from 2 to 4 in MoS_2_. **f** Circularly polarized PL for MoS_2_/WS_2_ under magnetic of 7 T (top), 0 T (middle), −7 T (down) respectively. The excitation power was around 200 μW. **g** Linear fitting of splitting energy as a function of magnetic field to extract *g* factor. The error bars are from the fitting uncertainties of the PL peak energies

Figure 2a shows the top and side schematic of the representative 2H stacked MoS_2_/WS_2_ heterostructures, where Mo and S atoms of the upper MoS_2_ layer lie on top of the S and W of the bottom WS_2_ layer. The average interlayer distances are determined to be 3.156 Å. The calculated band structures of the heterostructures including the SOC is shown in Fig. 2b. More detailed information about first-principles calculations can be found in the method section. Via orbital analysis, it can be known that the conduction band minimum (CBM) and valence band maximum (VBM) at K point are predominantly from Mo-*4d* and W-*5d* orbitals. Although density functional theory (DFT) and related methods often underestimate the electronic bandgap, they can provide basic guidelines for distinguishing specific type of the heterostructures. Figure 2c demonstrates the band alignment diagram for the investigated MoS_2_/WS_2_ heterostructures at K valley. It shows typical type II heterostructure character with CBM and VBM located at MoS_2_ and WS_2_ monolayer, respectively. Note that the simulated energy gap for interlayer exciton is consistent with our observed peak at low energy regime of 1.4 eV (See Supplementary Fig. 5 for more details).

### Enhanced valley splitting in MoS_2_/WS_2_ heterostructures

Due to the type II band alignment of MoS_2_/WS_2_ heterostructures, electrons and holes would separate in different layers through ultrafast charge transfer (Fig. 2d), leading to the *n*-doping and *p*-doping for MoS_2_ and WS_2_, respectively. Since the spin-splitting for the conduction band of MoS_2_ is relatively small (Δ_*CB*_ ≈ 3 me*V*)^53–55^, the ultrafast charge transfer induced *n*-doping in MoS_2_ could enhance the many-body Coulomb interactions strongly. As a result, the optical transitions of MoS_2_ will deviate significantly from the simple non-interacting particle picture and possess a largely enhanced magnetic response. To confirm this view, we performed circular polarization dependent PL measurements under an out-of-plane magnetic field (Faraday geometry) with 532 nm (2.33 eV) excitation laser at 4.2 K. The middle image in Fig. 2f shows the polarized PL for co-circularly polarized detection under right-circularly (*σ*^+^, blue curve) and left-circularly (*σ*^−^, red curve) polarized light excitation in the absence of magnetic field. It is known that, the *σ*^+^/*σ*^−^ can selectively excite the electrons in K/−K valley. There is no obvious energy shift at zero magnetic field due to the energy degeneracy of these two valleys. The two peaks located at 1.94 eV and 2.08 eV correspond to the direct band transition for monolayer MoS_2_ and WS_2_, respectively. Note that the interactions of the optically excited excitons with the surrounding Fermi sea of electrons would give rise to complex many-body states, such as trions, biexcitons and Fermi polarons^27,56–58^. Here we refer to them as the direct optical transitions simply. In contrast, at high field of ±7 T, the valley degeneracy is broken with higher energy for *σ*^+^/*σ*^−^ component at −7 T (bottom in Fig. 2f)/7 T (top in Fig. 2f). The valley splitting energy can be measured from the excitonic energy difference from the *σ*^+^ and *σ*^−^ polarized emission. It can be observed obviously in Fig. 2f that the valley splitting energy for MoS_2_ is much larger than that of WS_2_. Zeeman splitting energy as a function of magnetic field is plotted in Fig. 2g. According to Δ*E* = *gμ*_*B*_*B*, *g* factor for MoS_2_ and WS_2_ in heterostructures, extracted by linear fitting, can be calculated to be $g_{{\mathrm{MoS}}_{\mathrm{2}}} = 11.03 \pm 0.3$ and $g_{{\mathrm{WS}}_{\mathrm{2}}}^{} = 4.93 \pm 0.12$, respectively.

**Fig. 3:**
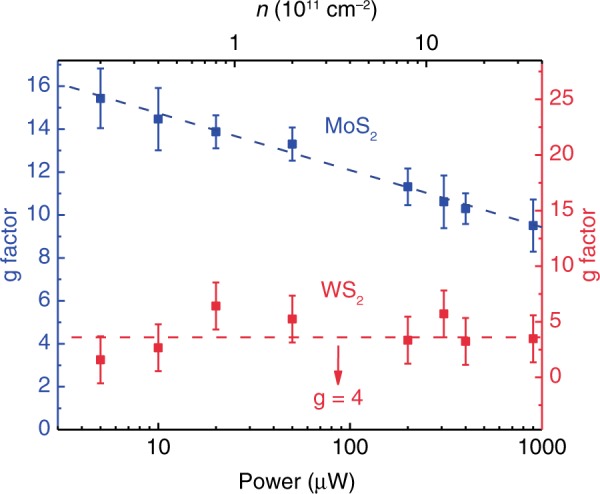
Excitation power dependence of the *g* factor for MoS_2_ and WS_2_. The error bars are from the fitting uncertainties of the PL peak energies. The x axis shows both the excitation laser power (bottom x axis) and the corresponding electron doping density (top x axis). The *g* factor value of MoS_2_ (WS_2_) corresponds to left blue (right red) y-axis. The blue (red) dash lines are guidelines for the evolution trend of *g* factor of MoS_2_ (WS_2_)

For WS_2_, the *g* factor is ~4 which is consistent with the previous results^59^. In stark contrast, *g* factor for the optical transition of MoS_2_ is increased dramatically and clearly violates the simple non-interacting independent particle picture. Such strong enhanced *g* factor for the optical transition of MoS_2_ can be understood as a combination of the relatively small spin-splitting for the conduction band of MoS_2_ and electron-doping of MoS_2_ induced by ultrafast charge transfer (Fig. 2e). First, the spin-splitting for the conduction band of MoS_2_ is only 3 meV^53–55^, the corresponding electron density, that is required to dope electrons into the upper conduction band, is ~2.5 × 10^11^ cm^−2^, an order of magnitude smaller than that for WSe_2_ (refer to Supplementary Discussion for more calculation details). Second, TMDCs usually possess a high density of sulfur vacancies up to 10^13^ cm^−2^, leading to electron-doping ~ 10^11^ cm^−2^^59^. Due to the large built-in interfacial electric field in type II MoS_2_/WS_2_ heterostructures, most of the electrons stemmed from sulfur vacancies of both MoS_2_ and WS_2_ will be localized in MoS_2_, resulting in a high electron-doping. Therefore, the Fermi level of MoS_2_ in MoS_2_/WS_2_ heterostructures is basically located in the spin-split upper conduction band, which can be clearly seen from the results of electric measurement (refer to Supplementary Fig. 3 for more details). Crossing the spin-polarized upper conduction band would lead to the change of spin-valley degeneracy (*l*_*s*_*l*_*v*_) from 2 to 4, as illustrated in Fig. 2e. For the exchange interaction, it is dependent on the spin-valley degeneracy strongly. And the strength of the exchange interaction is encoded in a dimensionless parameter $r = \sqrt {\frac{{l_sl_v}}{2}} r_s$, where the Wigner-Seitz parameter $r_s = \frac{1}{{\sqrt {\pi n} }}\frac{1}{{a_B}}$ denotes the strength of Coulomb repulsion energy and *a*_*B*_ is the effective Bohr radius. When the Fermi level crosses the upper conduction band, the doubling of spin-valley degeneracy could dramatically enhance the exchange interaction and causes the anomalously enhancement of *g* factor, as shown in Fig. 2g and Fig. 3. In this way, we could achieve strong magnetic response in MoS_2_ based on as-grown MoS_2_/WS_2_ heterostructures by intrinsic band alignment and ultrafast charge transfer doping.

### Optically controlled valley DoF in MoS_2_/WS_2_ heterostructures

Beyond significantly enhanced Zeeman splitting for MoS_2_ in MoS_2_/WS_2_ heterostructures, electron-doping in MoS_2_ can be controlled via varying the laser excitation power because of the ultra-long exciton recombination lifetime and the giant interfacial electric field. The manipulation of Coulomb repulsion energy and *g* factor through optical pumping provides an exceptional all-optical route towards tailoring the spin-valley properties in TMDCs heterostructures. Recently, tunable electron-doping caused by optical pumping has been demonstrated in bilayer WSe_2_ under an out-of-plane electric field^60^. Note that the band alignment of bilayer WSe_2_ under an out-of-plane electric field also belongs to type-II, being akin to the MoS_2_/WS_2_ heterostructures. Since the interfacial electric fields in MoS_2_/WS_2_ heterostructures is larger than the maximum applied electric field in bilayer WSe_2_, we can estimate the electron-doping caused by optical pumping in MoS_2_/WS_2_ heterostructures via using the results of the reference^60^ under the largest electric field, which is ~0.4 × 10^10^ cm^−2^/μW. In this way, we can obtain the concentration of external electron-doping caused by optical pumping under different power. The *g* factor of MoS_2_ and WS_2_ in the heterostructure versus the excitation power (bottom *x* axis) and the corresponding electron doping density (top *x* axis) is depicted in Fig. 3. As the power increases, the *g* factor of MoS_2_ goes through continuous decrease from 15.4 to 9.5 (refer to Supplementary Fig. 2 for more details), while the *g* factor of WS_2_ oscillates around 4 which corresponds to the value of undoped samples in previous studies^56^. Note that the *g* factor evolution of MoS_2_ with electron-doping in our work is in good harmony with the recent transport measurements^61^. For the continuous and monotonical decline in *g* factor of MoS_2_, it can be understood as the electron-doping tuned by optical pumping. As mentioned above, the Fermi level of MoS_2_ has crossed the upper of spin-polarized conduction band even without the optical pumping. When increasing the excitation laser power, the enhanced electron-doping would further lift the Fermi level within the upper conduction band of MoS_2_ and induce the drop of Wigner-Seitz radius *r*_*s*_. Thus, both the strength of the exchange interaction and Coulomb repulsion energy decrease, leading to decrease of *g* factor of MoS_2_ (Fig. 3). With the help of in-situ optical doping and efficient charge transfer in heterostructures, it could provide a unique picture of strong Coulomb interaction of 2D Dirac Fermions and a firm basis for the development of multi-bit optical computing. Such correlation between the magnetic, optical and valley coupling is important for potential applications of valley qubit for quantum computing, as the possibility of ultrafast generation and manipulation the valley pseudospin by coupling to photons. As for WS_2_, though it is strongly *p*-doped based on the band alignment, the large spin-orbit coupling energy (~400 meV) in valence band prevents the spin-valley degeneracy (*l*_*s*_*l*_*v*_) for holes to be considerably tuned. Thus, the valley spitting of WS_2_ in heterostructure is relatively immune to the excitation power.

### Effective magnetic control of valley polarization

**Fig. 4:**
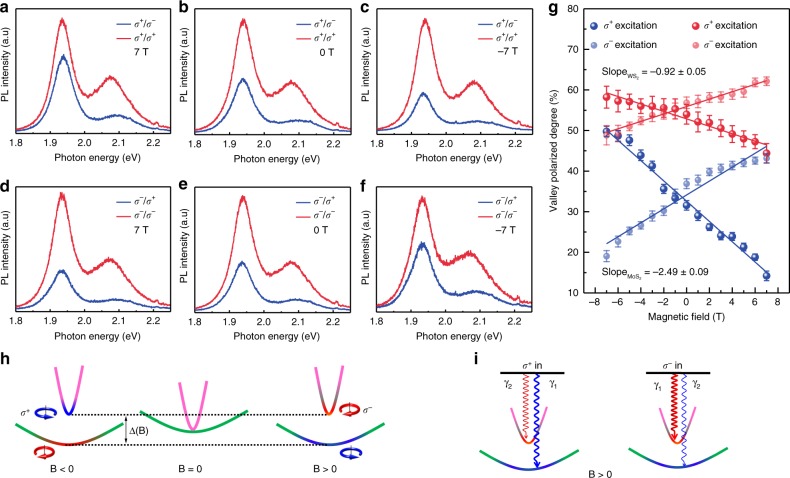
Magnetic control of valley polarization in MoS_2_/WS_2_ heterostructures. **a**–**f** Polarization-resolved photoluminescence under *σ*^+^ excitation (**a**–**c**) and *σ*^−^ excitation (**d**–**f**) for magnetic fields of 7 T (**a**, **d**), 0 T (**b**, **e**), and −7 T (**c**, **f**). **g** Degree of valley polarization for fundamental optical transitions of MoS_2_ and WS_2_. The error bars are from the fitting uncertainties of the PL peak intensities. **h** Dispersions of exciton energy spectrum with and without a magnetic field. Magenta and green represent the superposition of *σ*^+^ and *σ*^−^, and blue (red) denotes *σ*^+^ (*σ*^−^). **i** Exciton formation with valley-conserving process (*γ*_1_) and valley-flipping process (*γ*_2_) under *σ*^+^ (left) and *σ*^−^ (right) excitation for positive magnetic field

The strongly enhanced Lande *g* factor and valley splitting in MoS_2_/WS_2_ heterostructures, in principle, would enable the manipulation of the valley polarization effectively through magnetic field. To confirm this view, we measured the magnetic field dependent valley polarization of the direct optical transition in MoS_2_/WS_2_ heterostructures under both *σ*^+^ and *σ*^−^ excitations (Fig. 4a–f). The degree of valley polarization is defined as $\rho {\mathrm{ = }}\frac{{I_{{\mathrm{co}}} - I_{{\mathrm{cross}}}}}{{I_{{\mathrm{co}}} + I_{{\mathrm{cross}}}}}$ where *I*_co_ and *I*_cross_ are the intensities for co- and cross-circularly polarized detection, respectively. Figure 4g presents the magnetic field driven evolution of valley polarization for the direct optical transitions of both MoS_2_ (blue) and WS_2_ (red) in MoS_2_/WS_2_ heterostructures. It can be seen clearly that the valley polarizations of both MoS_2_ and WS_2_ are linear in magnetic field, with a negative (positive) slope for *σ*^+^ (*σ*^−^) excitation. Strikingly, the slope for the optical transitions of MoS_2_ (2.49) is obviously larger than that of WS_2_ (0.92), indicating that the valley DoF of MoS_2_ in MoS_2_/WS_2_ heterostructures can be manipulated more easily by magnetic fields. These phenomena can be understood as the combination of magnetic tuning of the dispersion of valley excitons and strongly enhanced Lande *g* factor in MoS_2_ induced by the enhanced exchange interactions. Due to exchange interactions between electrons and holes, the dispersion of valley exciton splits into two branches: the upper (lower) branch with a steep (smooth) dispersion^18^, as shown in Fig. 4h. For the zero-field case, the two branches touch at **k** = 0 and the two degenerate eigenstates are the excitons of K and −K, emitting *σ*^+^ and *σ*^−^ circularly polarized light, respectively (Fig. 4h, middle). Under a finite magnetic field, the degeneracy is lifted, opening a gap Δ(B) = *gμ*_B_B^18^. Since the steeper dispersion of upper branch requires much smaller momentum transfers when exciton is scattering into the light cone, it will facilitate the formation of exciton relative to the lower branch. For positive magnetic fields, the center of the upper (lower) branch is the −K (K) valley exciton (Fig. 4h, right). Thus, valley-conserving rate *γ*_1_ (valley-flipping rate *γ*_2_) under *σ*^+^ excitation would possess a smaller (larger) value than that for *σ*^−^ excitation, leading to a smaller valley polarization for *σ*^+^ excitation under positive magnetic fields (Fig. 4i). Similarly, it can be known that valley polarization under *σ*^+^ excitation would yield a larger value than that under *σ*^−^ excitation for negative magnetic field. For the slope which represents the ability of the magnetic field to tune the dispersion of valley excitons, it is determined by the gap Δ(B) between the upper and lower branches induced by the valley Zeeman effect. Due to the strongly enhanced valley magnetic response for MoS_2_ induced by strongly enhanced exchange interaction, the gap Δ(B) is much larger for MoS_2_ than that of WS_2_ (as illustrated in Fig. 2d). Therefore, we would observe a much larger slope for the optical transitions of MoS_2_ in Fig. 4g. This different magnetic response indicates the potential applications in storing multiple valley indexes as information bits with magnetic writing and optical readouts, opening exciting possibilities for valleytronic memory devices.

## Discussion

To summarize, we demonstrate the strongly enhanced valley Zeeman splitting and magnetic tuning of polarization in MoS_2_/WS_2_ heterostructures through all-optical logic gates. The strong enhancement of valley magnetic response is due to the doubling of the spin-valley degeneracy and strongly enhanced electron-electron exchange interactions induced by ultrafast charge transfer. In addition, we show that significantly enhanced valley susceptibility can be tuned monotonically by optical pumping, offering a unique strategy for valleytronic applications. This heterostructure based approach designates a novel way for future exploration in strongly charged exciton polariton and spin-polarized Landau levels in TMDCs. Our results provide compelling evidence for band alignment induced intrinsic many-body interaction effects and establish a fertile ground for exploring the strongly correlated phenomena and ferromagnetic instability of massive Dirac electrons based on all-optical logic gates.

## Methods

### Growth of vertically stacked MoS_2_/WS_2_ heterostructures

Growth process was carried out in home-made three-zone CVD system with 1-inch quartz tube using WO_3_ (Alfa Aesar 99.999%), MoO_3_ (Alfa Aesar 99.999%) and S (Alfa Aesar 99.9%) powder as precursors. Each of the temperature zone was heated to preset values at a rate of 25 °C/min and kept stable for 20 min prior to growth. The precursor powders were pre-placed outside of the furnace and rapidly loaded from outside into each zone to start the growth. During the growth, Argon was used as carrying gas at a flow rate of 130 sccm and the vacuum pressure was kept at 0.7 Torr. The typical temperature for each three zones are 115 °C, 560 °C, and 800 °C, respectively for MoS_2_ growth.

### Optical characterization and magneto-PL measurements

The Raman and PL spectra in Fig. 1c, d were measured using a confocal microscope system (WITec, Alpha 300) with a frequency doubled YAG 532 nm laser at room temperature. For the magneto PL, we used a custom-designed attocube confocal micro-PL spectroscopy/imaging system with sample stage consisting of x-, y-, and z- axis positioners and a x-, y- scanner. The excitation light was a continuous-wave laser of 532 nm through a ×50 objective (laser spot size ~1 μm diameter). As for the configuration of circularly resolved PL, we inserted a quarter wave plate along incident laser to obtain circular polarized light excitation. The accuracy of the circular polarization degree for our setup was >93.0%. The emission light went through the same quarter wave plate plus a linear polarizer to be filtered by left/right helicities before CCD collection. A 600 lines/mm grating was used for PL measurements. Magnetic fields in the range of +7 T to –7 T were applied perpendicular to the plane of the heterostructure and monolayer samples.

### First-principles calculations

Our calculations are performed based on the density functional theory (DFT) as implemented in the Vienna Ab-initio Simulation Package (VASP)^62^. We utilize the generalized gradient approximation (GGA) with the Perdew-Burke-Ernzerhof (PBE)^63^ realization for the exchange-correlation potential. To properly take the vdW interactions into consideration, the recent developed vdW density functional (i.e., SCAN + rVV10)^64^ is employed. The plane-wave cutoff energy is set to be 400 eV. The Monkhorst-Pack *k*-point mesh of size 21 × 21 × 1 is used for the Brillouin zone (BZ) sampling^65^. The thickness of the vacuum layer is set to be 25 Å, which is adequate to simulate two-dimensional materials. The convergence criteria of the total energy and the force on each atom were set to 10^−5^ eV and 10^−3^ eV Å^−1^, respectively. The SOC effect is included for the band structure calculation.

## Supplementary information

Supplementary Information

## Data Availability

The data that support the findings of this study are available from the corresponding authors upon request.
