# Supplementary material for: Enhancing and controlling valley magnetic response in MoS2/WS2 heterostructures by all-optical route
Source: Nat Commun. 2019 Sep 17;10:4226. doi: 10.1038/s41467-019-12128-2 (PMC6748949; doi:10.1038/s41467-019-12128-2)
Supplement: Supplementary file 1 — Supplementary Information [file 41467_2019_12128_MOESM1_ESM.pdf]

Supplementary Information

Enhancing and controlling valley magnetic response in MoS_2/WS_2 heterostructures by all-optical route

Jing Zhang[1†], Luojun Du[2,3†], Shun Feng[1†], Run-Wu Zhang[4,5], Bingchen Cao[1], Chenji Zou[1], Yu Chen[1], Mengzhou Liao[2], Baile Zhang[1], Shengyuan A. Yang[4], Guangyu Zhang[2,6*] and Ting Yu[1*]

1. *Division of Physics and Applied Physics, School of Physical and Mathematical Sciences, Nanyang Technological University, Singapore 63737, Singapore*
2. *CAS Key Laboratory of Nanoscale Physics and Devices, Institute of Physics, Chinese Academy of Sciences, Beijing, 100190, China*
3. *Department of Electronics and Nanoengineering, Aalto University, Tietotie 3, FI-02150, Finland*
4. *Research Laboratory for Quantum Materials, Singapore University of Technology and Design, Singapore 487372, Singapore*
5. *Key Lab of advanced optoelectronic quantum architecture and measurement (MOE), Beijing Key Lab of Nanophotonics & ultrafine Optoelectronic Systems, and School of Physics, Beijing Institute of Technology, Beijing 100081, China*
6. *Songshan Lake Materials Laboratory, Dongguan, Guangdong 523808, China*

† These authors contribute equally to this work.

Corresponding authors: gyzhang@iphy.ac.cn, yuting@ntu.edu.sg

Supplementary Discussion

Calculations of required electron density to lift Fermi level to cross spin-polarized upper conduction band. For the conduction band of MoS_2, the dispersion relation is as follows:

$$E = E_c + \frac{\hbar^2}{2m^*}(k_x^2 + k_y^2) \qquad (1)$$

where m^* is the effective mass and 0.4 times the free electron mass.

Using the periodic boundary conditions, quantized values k_x and k_y are:

$$k_x = n_x \frac{2\pi}{L_x} \qquad k_y = n_y \frac{2\pi}{L_y} \qquad (2)$$

where L_x and L_y are the dimensions of the sample, n_x and n_y are integers.

Therefore, the area in the k_x-k_y plane 'occupied' by an individual state is given by:

$$\frac{2\pi}{L_x} \times \frac{2\pi}{L_y} = \frac{4\pi^2}{L_x \times L_y} \qquad (3)$$

The total number of states $N_T(E)$ for the area enclosed by the circle $\pi \boldsymbol{k}^2$:

$$N_T(E) = \frac{\pi \boldsymbol{k}^2}{\frac{4\pi^2}{L_x \times L_y}} = \frac{\boldsymbol{k}^2(L_x \times L_y)}{4\pi} \qquad (4)$$

The density of states per unit area per unit energy is given by:

$$N(E) = \frac{1}{L_x \times L_y}\frac{dN_T(E)}{dE} = \frac{m}{2\pi\hbar^2} \qquad (5)$$

For MoS_2, the spin-splitting for the conduction band Δ_{cB} is 3 meV.

Thus, corresponding electron density, that is required to dope electrons into the upper conduction band, is:

$$n = N(E) \times \Delta_{cB} = 2.5 \times 10^{11}/cm^2 \qquad (6)$$

We also calculated the electronic band structures for monolayer MoS_2 and WS_2 (with SOC included), as shown in Supplementary Figure 1. It can be observed that the SOC effect causes the spin splitting of both VBM and CBM level at the K-symmetry point, while energy splitting for valence band is much larger than that of conduction band for both MoS_2 and WS_2. Supplementary Figure 1b and Supplementary Figure 1d are magnified energy splitting of the CBM at K point, which can be determined to be 3 meV and 30 meV for monolayer MoS_2 and WS_2, respectively.

Supplementary Figures

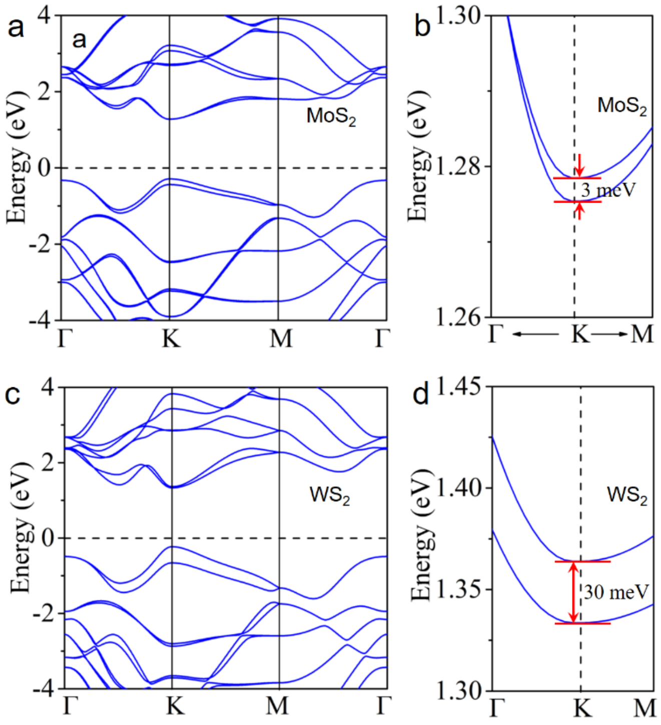

Supplementary Figure 1. (a) Band structure of monolayer MoS_2. (b) Magnified energy splitting of the MoS_2 CBM at K point. (c) Band structure of monolayer WS_2. (d) Magnified energy splitting of the WS_2 CBM at K point.

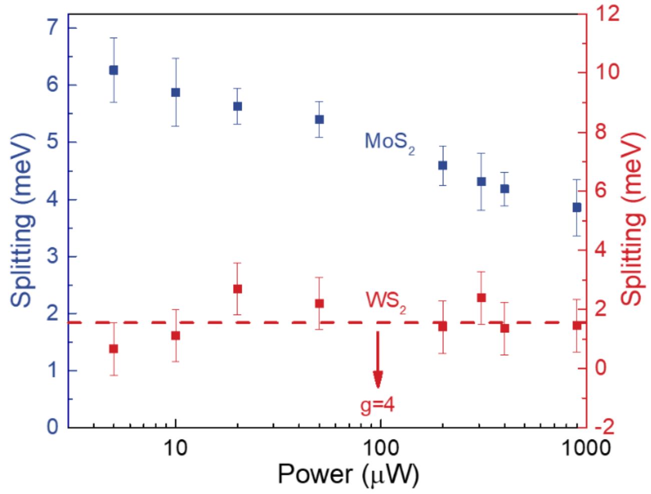

Supplementary Figure 2. Excitation power dependence of the valley Zeeman splitting energy for MoS_2 and WS_2. The error bars are from the fitting uncertainties of the PL peak energies. The splitting value of MoS_2 (WS_2) corresponds to left blue (right red) Y-axis.

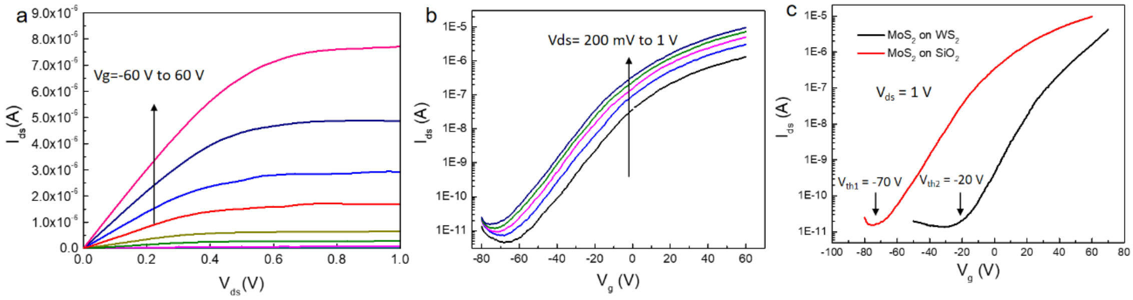

Supplementary Figure 3. (a) Typical output curves for MoS_2 devices on WS_2 with gate voltage (V_g) sweeping from -60 V to 60 V. (b) Typical transfer curves for MoS_2 devices on WS_2 with source-drain voltage (V_{ds}) from 200 mV to 1 V. (c) Transfer curve comparison for MoS_2 devices on WS_2 and SiO_2, respectively.

We have fabricated back-gated field effect transistors based on single-crystalline MoS_2 domains on WS_2 and Si/SiO_2 substrates. Supplementary Figure 3a and 3b are output and transfer curves of a typical MoS_2 device on WS_2, showing a typical *n*-type transistor behavior with threshold voltage located at around -70 V. We calculated the electron mobility of MoS_2 FET device based on the standard transistor model. The resulted mobility and on/off current ratio at room temperature are ~40 cm^2/Vs and 10^6, respectively. We also compared the transfer curves of MoS_2 domains on WS_2 and SiO_2 in Supplementary Figure 3c. It can be clearly observed that the threshold voltage shifting from -20 V on the SiO_2 to -70 V on WS_2 domains. As is known, the position of threshold voltage reflects the doping level of device, which may come from (1) the intrinsic *n* doping from the sulfur vacancies; (2) the doping introduced by surface contaminations during device fabrication process; or (3) the doping induced by the underneath WS_2. Due to the same growth conditions and device micro-fabrication processes, (1) and (2) should not change dramatically. The shift of threshold voltage and enhanced electron doping can be attributed to the electron transfer from WS_2 to MoS_2, which is consistent with our optical results in the manuscript.

Here, we can roughly estimate the electron doping density based on parallel-plate capacitor model: $n_{2D} = C_{ox}\Delta V_{bg} / e$; where $C_{ox} = \varepsilon_0\varepsilon_r / d_{ox}$, $\varepsilon_0 = 8.85\times10^{-12} Fm^{-1}$, $\varepsilon_r = 3.9$, $e = 1.6\times10^{-19} C$. The charge transfer between WS_2 and MoS_2 makes

prominent contributions to the threshold voltage shift $\Delta V_{bg} = |V_{th1} - V_{th1}| = 50V$, giving typical n doping concentration variation of $\Delta n_{2D} = 3.6\times10^{12}\,cm^{-2}$ which is larger than the required electron doping density required to dope electrons into the upper conduction band. Therefore, the Fermi level of MoS_2 in MoS_2/WS_2 heterostructures is basically located in the spin-split upper conduction band.

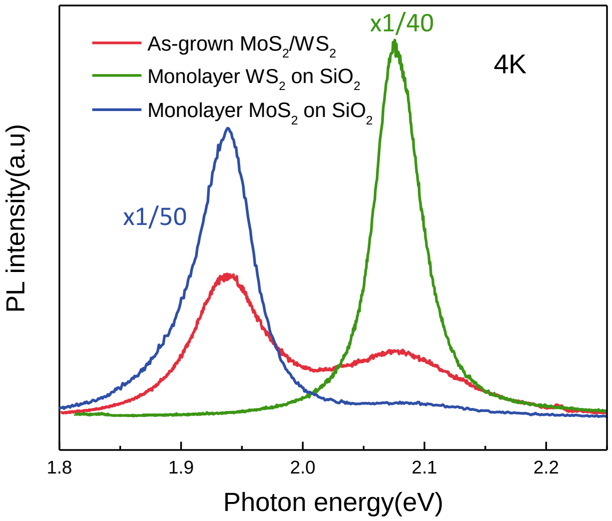

Supplementary Figure 4. PL spectra for monolayer WS_2, MoS_2 and MoS_2/WS_2 heterostructures at 4.2K.

Supplementary Figure 4 shows the PL spectra for monolayer MoS_2, WS_2 and heterostructures at 4.2 K. Despite the energy difference of excitons, the PL spectra at 4.2 K are in good harmony with that at room temperature. Due to ultrafast interlayer charge transfer, PL intensity in heterostructures is quenched by a factor of 50 (40) compared with MoS_2 (WS_2).

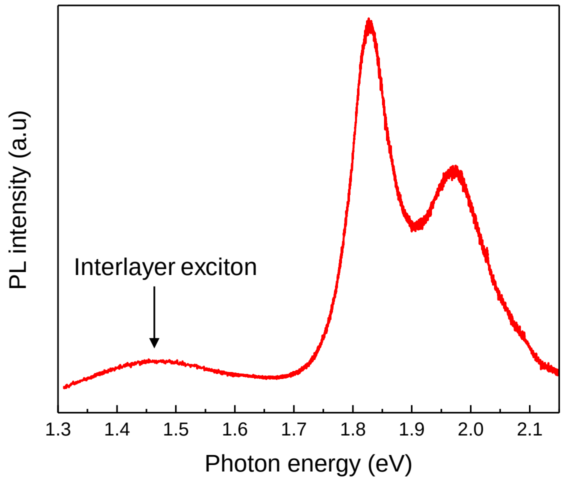

Supplementary Figure 5. PL spectra for as-grown MoS_2/WS_2 heterostructures at room temperature.

Supplementary Figure 5 shows the PL spectrum of MoS_2/WS_2 heterostructures at room temperature with a large energy range. In addition to the intralayer excitons at high energy regime, we can also observe a broad peak located at 1.45 eV. This new low energy peak shows good agreement with interlayer exciton obtained via theoretical calculation (Fig. 2c in the main text).

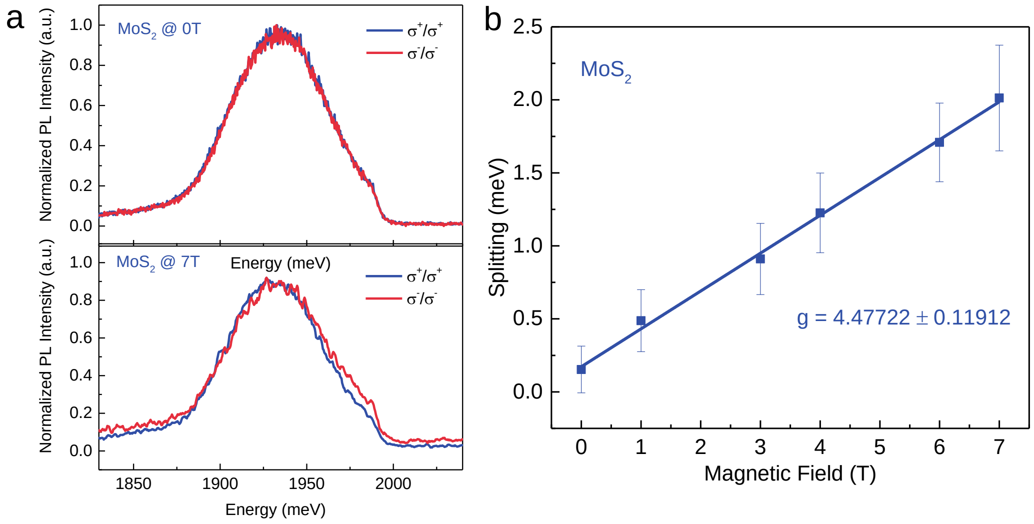

Supplementary Figure 6. Valley Zeeman splitting for isolated monolayer MoS_2 at 4.2 K. (a) Circularly polarized PL for isolated monolayer MoS_2 flake under magnetic of 0 T (top) and 7 T (bottom). (b) Linear fitting of splitting energy as a function of magnetic field to extract g factor.

Supplementary Figure 6a shows the circularly polarized PL for isolated monolayer MoS_2 flake with out-of-plane magnetic field of 0 T (top panel) and 7 T (bottom panel), respectively. There is no obvious energy shift for right-circularly (σ^+, blue curve) and left-circularly (σ^-, red curve) polarized light excitation without magnetic field. The valley Zeeman splitting can be clearly observed from σ^+ and σ^- polarized emission at 7 T. Valley splitting energy as a function of magnetic field is plotted in Supplementary Figure 6b. The extracted g factor can be determined to be around 4.47 ± 0.12 by linear fitting. This result is closely in line with previous reports of undoped TMDs, which can be understood in non-interacting regime.
